# Exploring the relationship of sleep, cognition, and cortisol in sickle cell disease

**DOI:** 10.1016/j.cpnec.2022.100128

**Published:** 2022-03-04

**Authors:** Melanie Kölbel, Fenella J. Kirkham, Ray K. Iles, Hanne Stotesbury, Elizabeth Halstead, Celia Brenchley, Sati Sahota, Dagmara Dimitriou

**Affiliations:** a Clinical Systems Neuroscience Section, UCL Great Ormond Street Institute of Child Health, London, UK; b Sleep Education and Research Laboratory, UCL Institute of Education, London, UK; c Clinical and Experimental Sciences, University of Southampton, Southampton, UK; d NISAD, Medicon Village, SE-223 81, Lund, Sweden; e Laboratory of Viral Zoonotics, Department of Veterinary Medicine, University of Cambridge, Madingley Road, Cambridge, CB3 0ES, UK

**Keywords:** Sickle cell, Cortisol, Cognition, Sleep, Sleep disorders, Actigraphy, SCD, Sickle cell disease, OSA, Obstructive sleep apnoea, PLMS, Periodic limb movement syndrome, SDB, Sleep disordered breathing, CAR, Cortisol awakening response, DCR, Diurnal cortisol ratio, PSI, Processing Speed Index, WMI, Working Memory Index, PRI, Perceptual Reasoning Index, VCI, Verbal Comprehension Index, FI, Fragmentation index, SOL, Sleep onset latency, SES, Socioeconomic status, NCP, Normal cortisol profile, FCP, Flat cortisol profile

## Abstract

**Background:**

Neurocognitive impairment is common in people with Sickle Cell Disease (SCD) and evidence is accumulating that sleep disturbances play a role. The interaction between cortisol and sleep in the general population is associated with cognition as well as general wellbeing but there are few data in SCD. We aimed to understand the relationship between cortisol and sleep in individuals with SCD and explored associations with cognition.

**Methods:**

Forty-five participants of black heritage (SCD: N = 27, 9–29 years, 16 females; Controls: N = 18, 11–25 years, 13 females) were recruited from the community between 2018 - 2020. Participants completed standardized questionnaires about their sleep behaviour and wore actigraphy MotionWatch8 for 7 nights to assess nocturnal sleep patterns. Salivary cortisol samples were taken on wakening and 3 times after 14:00. Cognition was assessed using the Wechsler Intelligence Scales for children and adults.

**Results:**

People with SCD took longer to fall asleep and experienced greater wake bouts, mobile minutes and fragmented sleep compared to controls. Although non-significant, people with SCD experienced lower morning cortisol, with a flattened diurnal cortisol ratio compared to controls. Interestingly, SCD participants, but not controls, with low diurnal variation scored lowest on processing speed (PSI) and perceptual reasoning index (PRI). A moderator analysis revealed that the effect of morning cortisol and diurnal cortisol ratio on PRI by group health (i.e., SCD and healthy controls) depended on sleep quality.

**Discussion:**

Sleep and cortisol may play a crucial role in the expression of cognitive difficulties seen in SCD. This should be considered for the development of interventions to optimise cognitive functioning and sleep. This, in turn, could positively impact on secretion of cortisol and general health in SCD.

## Highlights

• We examined sleep patterns, cortisol and cognition in 27 participants with Sickle Cell Disease (SCD) and 18 healthy controls.
• People with SCD had lower cognitive scores compared to healthy controls.
• People with SCD took longer to fall asleep, had greater wake bouts, mobile minutes, and fragmented sleep.
• People with SCD experienced a flattened diurnal cortisol profile.
• Sleep disturbances might interfere with diurnal cortisol rhythm and contribute to lower cognitive scores .

## Graphical abstract

**Figure:**
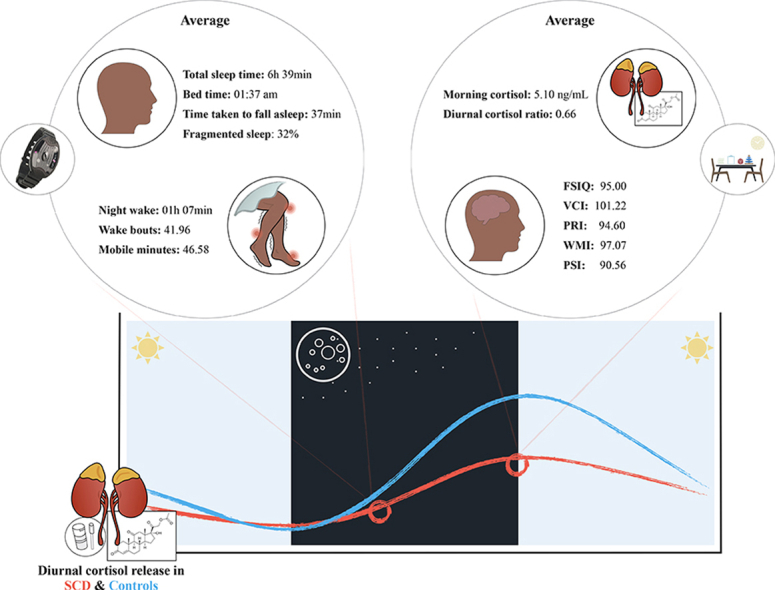

## 1 Introduction

The most common recessively inherited red blood cell disorder is sickle cell disease (SCD), in which the majority of the beta-globin within red blood cells is the sickle variant, which alters the oxygen binding characteristics of the alpha-beta, alpha-beta tetrameric complex of haemoglobin. Under hypoxic condition, the sickle mutant beta-globin undergoes beta-beta globin polymerisation into a rod-shape, which deforms the entire red blood cell into a sickle-shape: Whilst the red cells ability to carry oxygen is reduced, the structural deformation makes the red blood cells more rigid and less able to deform and pass through restricted small blood vessels [1].

The prevalence of sleep difficulties and disorders are high in young populations with SCD, who are mainly of black African heritage and therefore already predisposed [2]. Parents reported that sleep onset insomnia is already present in 13% of 1-6-year-olds with SCD, increasing to 20% in 7–12-year-olds [3]. Sleep disorders, such as obstructive sleep apnoea (OSA), are diagnosed in 69% of individuals under 18 years old referred for symptoms of sleep disordered breathing (SDB) [4], and depending on definition around 20–40% of an unselected population [5]. At least a quarter of children, and up to 70% of adults, with SCD undergoing polysomnography are diagnosed with periodic limb movement syndrome (PLMS) [4,6]. Actigraphy, previously used in SCD research [7], is a cost-effective, ecologically valid, non-invasive sleep measure, which has been validated and correlates well with polysomnography [8]. Sleep fragmentation, one of the measures collected by actigraphy and an indication of sleep quality, is shown to be higher in individuals with SCD (4–23 years) [9] as well as male adolescents of black heritage (aged 14–19 years) [10].

In the general population, several nights of sleep deprivation affects cognition, impacting on attention and working memory [11,12]. Lower performance on working memory tasks were observed in children (8–12 years) with OSA [13], as well as adults with fragmented sleep and sleep disordered breathing (SDB) [14], or restless leg syndrome [15]. There is emerging evidence that various aspects of sleep, including duration, fragmented sleep (i.e., frequency of arousals) and exposure to hypoxia are associated with lower processing speed and executive functioning in individuals with SCD [16,17].

Cognitive difficulties in SCD emerge at an early developmental stage and continue into adulthood, with low scores in childhood in those with and without clinical stroke and silent infarction [18,19]. Recent research has therefore been focused on preventing infarction, with little investment in any involvement of other modifiable risk factors for cognitive differences observed in people with SCD compared with controls, such as exposure to hypoxia, sleep disorders or disadvantaged socioeconomic status (SES), which may also play a role. For example, low SES and sleep deprivation were predictive of slower reaction time in 8–9 year old children of African American heritage [20].

Children and adolescents with SCD (4–23 years old) who exhibited behavioural signs such as daytime sleepiness (i.e., difficulty staying awake during the day) had lower sleep quality, and experienced more SDB, night awakenings and movement at night [9]. In the general paediatric population, sleep fragmentation as a consequence of SDB can promote hypothalamic–pituitary–adrenal axis (HPA) dysfunction with an increase in cortisol release, resulting in daytime sleepiness [21,22]. However, there are few data in children with SCD.

Cortisol has metabolic effects and is related to sleep quality [23]. Released by the adrenal cortex, cortisol follows a circadian rhythmicity with high concentration after awakening, followed by a rapid decline with the lowest concentration around sleep onset [24]. Metabolic effects are greatest during arousal from sleep with a dramatic rise to a maximum in blood cortisol to raise energy production by the body, replenish and maintain glycogen stores and also match the needs of the active body state.

After one night of sleep deprivation, dysregulation of the HPA axis, with a flattened morning cortisol profile [25], has been shown in healthy young adults (mean age 23 years).

Interestingly, repeated apnoeic events during sleep may also promote a flattened cortisol awakening response (CAR), as shown in an adult population with SDB (40–60 years) [26] and in children (3–13 years) with OSA [27].Individuals with SCD generally experience lower plasma cortisol levels compared to healthy controls [[28], [29], [30]] and in one study, adrenal insufficiency was identified in 19.4% of individuals with SCD [31]. However, higher concentrations have been observed during episodes of acute illness (e.g. vaso-occlusive pain crises) [32]. There are few data on any relationship between cortisol levels and sleep duration or quality in SCD.

Sleep deprivation influences the diurnal secretion of cortisol and has a parallel effect on neurocognitive function in the adult general population. For example, after 25 h of wakefulness healthy adults (mean age 29 years) had a flattened cortisol slope and a slower response speed for neutral words and positive targets [33]. Research in other populations has shown that an altered cortisol response is linked to reduced cognitive functioning [34,35]. For example, a greater CAR slope was related to better episodic memory, while higher morning cortisol was associated with better working memory in one study of healthy adults (23–79 years) [36]. Tsui et al. [35] found that decreased AM:PM cortisol ratio predicted worse later life cognitive function and verbal fluency while another study found higher morning cortisol associated with better processing speed and executive functioning in community-dwelling elderly adults [37]. Perceptual reasoning skills were better in undergraduate students who experienced high cortisol levels during cognitive testing with the Wechsler Intelligence Scale [38]. Adverse life events often accumulate in families of disadvantaged SES and a study has shown that their children (6–7 years old), not only experience more stress, which is associated with a flatter CAR, but also perform poorly on tasks measuring memory performances [39]. However, there are few data on diurnal cortisol variation and cognitive function in children, adolescents, or young adults or in individuals with SCD.

Daytime sleepiness [9] working memory, perceptual reasoning and processing speed difficulties reported in individuals with SCD [19,40], could be associated with chronic sleep disturbances and altered cortisol responses. Only a few studies have examined the relationship between sleep and cognition in SCD [16,17,41]. Of these, the majority have examined the impact of SDB on cognition [16,17]. Prior studies have also not prioritized the inclusion of ethnicity-matched healthy controls. To our current knowledge, there is no current research in SCD that has investigated the relationship between diurnal variation in cortisol levels and cognition using quantitative measures. We hypothesise that (1) Young individuals with SCD experience lower morning cortisol and a flatter cortisol profile than their age- and race-matched controls (2) Cortisol profiles are related to sleep quality using actigraphy measures in young individuals with and without SCD (3) Cognitive performance on the Wechsler scales is related to sleep quality and cortisol profiles. The aim of the current study was therefore to investigate sleep using diaries and actigraphy, cognitive performance from the Wechsler scales, and cortisol profiles in SCD with ethnicity-matched controls to explore the relationship between these profiles.

## 2 Methods

### 2.1 Participants

Individuals with SCD and healthy controls of black heritage aged 6–30 years were eligible for the study. All participants were recruited from the community in London (i.e., advertisement on Instagram, Twitter, through friends and family, as well as local advertisement in community centres and charity). The study was approved by the Research Ethics Committee (14475/001). Written informed consent was obtained from all participants and for children from their parent/guardian; the children also gave written assent.

### 2.2 Measures

#### 2.2.1 Questionnaires

Caregivers or participants were asked to fill in basic demographic information at the start of the study (i.e., age, sex, and post-code). The post-code based Index of Multiple Deprivation (IMD) provided an index of SES, as previously described [42]. There are seven domains that account for the IMD: income, employment, education, health, crime, barriers to housing & service, living environment [43]. The data are open-source, and downloadable from the UK Ministry of Housing, Communities & Local Government (http://imd-by-postcode.opendatacommunities.org/imd/2019). The index ranges from rank 1st (most deprived) to 32,844th (least deprived).

#### 2.2.2 Actigraphy

The MotionWatch8, manufactured by CamNtech, is a CE marked Class 1 medical device with FDA approval (K132764), which has very good validity. It monitors physical day and night-time movement with an internal miniature accelerometer [44]. The watch was worn for 7 nights on one week on the non-dominant wrist. The actigraphy collected movement samples in 30 s epochs. Extracted data included information on total sleep time (i.e. actual time asleep, categorized by wake/sleep categorisation), bedtime (i.e. time fallen asleep), sleep onset latency (i.e. the time between turning off the lights and falling asleep), wake time (i.e. time awake at night), wake bouts (i.e. contiguous periods of wake during the night), mobile minutes (i.e. time spend moving during the night), fragmentation index (i.e. degree of movement during the night, an indication of sleep quality), mean night activity per epoch (i.e. total movement count per 30s epoch during sleep) and central phase measure (i.e. midpoint of sleep in minutes: negative number reflects time before midnight). Data were analysed using the CamNtech Motionware software on a computer.

#### 2.2.3 Cortisol

Participants were asked to take four samples of their saliva during one weekday of the week during which they wore the actigraphy watch (i.e., not a weekend day) [45]. Research has shown that salivary levels correspond closely to blood samples [46]. Participants were given a collection protocol after they underwent training in the correct collection of their saliva sample. An oral fluid collector (OFC) (Soma Bioscience Ltd., Wallingford, UK) was used to collect the samples, consisting of a synthetic polymer swab designed to collect 0.5 mL of saliva, mixed with 3 mL of OFC buffer. This technique is stable at room temperature for several weeks and is unaffected by recent food and drink ingestion. The first sample was taken no later than 30 min after awakening, followed by one in the afternoon (2pm), evening (after 4pm) and at bedtime. Salivary cortisol was determined using an enzyme immunoassay (EIA) test kit (Soma Bioscience Ltd., Wallingford, UK), and read on an automated analyser (Tecan Nanoquant, Männedorf, Switzerland). The assay ranges for cortisol were 0.25–32.0 ng/mL. In the case of obtaining maximum values, samples were titrated and re-analysed. Cortisol profiles were assessed using all 4 values to define if each participant had a flat or normal cortisol profile compared to the individual group mean, since there are currently no reference values available. We also examined the raw morning cortisol and diurnal ratio (DCR) (PM/AM).

#### 2.2.4 Cognitive assessment

Pulse oximetry was used to measure daytime oxygen saturation (i.e., SpO_2_) on the day of cognitive assessment using a pulse oximeter. General cognitive abilities (Full Scale IQ) were assessed using the Wechsler Intelligence Scale for Children ≤15 years old (WISC-IV) [47] or the Wechsler Adult Intelligence Scale ≥16 years old (WAIS-IV) [48].

The assessment took place the same week of the sleep study and cortisol sampling (i.e., while wearing the Actiwatch). The FSIQ comprises the Processing Speed Index (PSI), Working Memory Index (WMI), Perceptual Reasoning Index (PRI) and Verbal Comprehension Index (VCI). The literature has shown strong correlations between the WAIS and WISC, allowing both to be used in the same analyses [49].

#### 2.2.5 Procedure

Participants were asked to monitor their sleep profiles for one week (i.e., 7 nights) with the Actiwatch and a sleep diary. During this time an appointment was made for the participant to attend their cognitive assessment. The salivary samples were taken on a weekday (i.e., not weekend).

### 2.3 Statistical analysis

Statistical analysis was performed using SPSS® version 26 (IBM Corporation, Armonk, NY, USA) for Mac®. Results are given in Mean ± SD unless otherwise stated. For each variable, normality and homogeneity of variance were assessed using the Shapiro-Wilk test. Cook's distance was used to identify potential outliers. Appropriate parametric or non-parametric tests were then chosen to compare variables between patients and controls, and to explore associations between variables. Raw actigraphy data were downloaded from CamNtech Motionware software and entered in Excel to create an Actogram of day and nighttime behaviour for mean activity per 30s epoch. Participants with incomplete data or missing data were excluded. The custom dialog box “PROCESS” for SPSS [50] was used to perform a three-way moderation analysis (model 3) to understand the moderation of cortisol on cognition by health status (SCD or control), which is moderated by sleep quality.

## 3 Results

### 3.1 Participant characteristics

Twenty-seven participants with SCD (HbSS, 16 females/11 males; Mean_age_ = 19.3 ± 5.2; 4 primaryschool-aged children, 9 adolescents, 14 young adults) and eighteen healthy controls (2 HbAS, 13 females/5 males; Mean_age_ = 19.4 ± 4.0; 1 primary school-aged child, 6 adolescents, 11 young adults) were eligible for inclusion in analyses. One participant was excluded from the sleep analysis due to non-compliance (i.e., only recorded 3 nights, which is less than the recommended 5 nights required for the sleep analysis [44]. Nine participants were excluded from the cortisol analysis due to incorrect sampling (*N* = 2 SCD, *N* = 1 control), missing values (*N* = 2 SCD, *N* = 2 controls), abnormal extreme value (*N* = 1 control) or forgetting to take the samples (*N* = 1 SCD). All had useable cognitive and sleep diary data, while 44 participants had useable sleep actigraphy data and 36 had useable cortisol data (Flowchart: Supplement Figure 1). No significant difference in relation to age, sex, or SES (*p* > 0.05) was observed between SCD participants and controls, or for SCD participants with (*N* = 10) and without Hydroxyurea prescription (*N* = 16). Participants with SCD had significantly lower SpO_2_ compared to controls (Table 1).

**Fig. 1:**
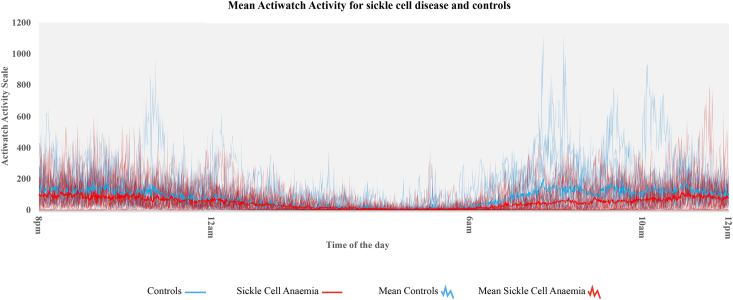
Day and nighttime activity as measured with actigraphy.

**Table 1:** Participant comparisons for general demographics, sleep profile, cognitive functions and cortisol.

	SCD	*CI 95%*	Controls	*CI 95%*	SCD + C	*Effect size*
**Demographics**						
*N*	27		18			
Sex	11 Male		5 Male		*p* > 0.05	
	16 Female		13 Female			
Black African British	23 (85.18%)		15 (88.24%)		*p* > 0.05	
Black Caribbean British	4 (14.82%)		2 (11.76%)			
Age in years	19.33 (5.16)	[17.29,21.37]	19.43 (3.99)	[17.44,21.41]	*p* = 0.94	0.02
	9.05–29.4		11–25.1			
SES	9159.52 (6688.62)	[6513.59,	8578.56 (6896.55)	[5148.98,	*p* = 0.49*	0.16
	1442–32371	11805.45]	3056–26063	12008.13]		
SpO2	96.75 (2.45)	[95.71,97.79]	99.15 (0.8)	[98.67,99.64]	*p* < 0.01*	1.22
	90–100		98–100			
**Sleep profile**						
*N*	26		18			
TST (hh:mm)	06:28 (01:06)	[06:01,06:54]	06:11 (00:54)	[05:44,06:38]	*p* = 0.69	0.28
	04:51–08:23		04:33–08:12			
Bed time (hh:mm)	01:02 (01:26)	[00:28,01:37]	00:52 (01:18)	[00:13,01:31]	*p* = 0.36	0.12
	21:01–03:24		22:57–03:58			
SOL (hh:mm)	00:48 (00:42)	[00:31,01:05]	00:35 (00:27)	[00:21,00:40]	*p* = 0.32*	0.15
	00:08–03:06		00:03–01:55			
Waketime (hh:mm)	08:36 (01:29)	[08:00,09:12]	07:58 (01:15)	[07:20,08:36]	*p* = 0.10*	0.10
	05:54–11:32		06:02–11:22			
Night wake (hh:mm)	01:04 (00:26)	[00:54,01:15]	00:57 (00:21)	[00:47,01:08]	*p* = 0.51*	0.25
	00:32–02:14		00:28–01:48			
Wake bouts	40.75 (13.54)	[35.29,46.22]	33.95 (7.74)	[30.11,37.81]	*p* = 0.04	0.62
	22–75		18–42.80			
Mobile minutes	45.22 (15.86)	[38.81,51.62]	35.21 (10.87)	[29.80,40.61]	*p* = 0.03	0.74
	20.30–77.80		17.20–53.80			
Fragmentation Index %	31.28 (9.28)	[27.53,35.03]	25.41 (7.08)	[21.89,28.93]	*p* = 0.02	0.71
	15.30–54.30		11.10–36			
Central Phase Measure	290.25 (78.81)	[258.42,322.08]	268.11 (73.67)	[231.47,304.74]	*p* = 0.35	0.29
	93.80–424.4		164.80–460.40			
**Sleep profile week**						
*N*	26		18			
TST (hh:mm)	06:24 (01:06)	[05:55,06:35]	06:01 (01:03)	[05:29,06:32]	*p* = 0.26	0.36
	04:12–08:19		02:58–08:17			
Bed time (hh:mm)	00:51 (01:28)	[00:15,01:26]	00:35 (01:21)	[23:55,01:16]	*p* = 0.26*	0.17
	21:06–03:27		22:16–03:41			
SOL (hh:mm)	00:51 (00:43)	[00:34,01:08]	00:38 (00:30)	[00:22,00:53]	*p* = 0.45*	0.11
	00:09–02:52		00:01–02:10			
Waketime (hh:mm)	08:35 (01:42)	[07:40,09:01]	07:55 (01:42)	[06:49,08:23]	*p* = 0.14	0.39
	05:51–11:46		06:01–12:01			
Night wake (hh:mm)	01:04 (00:27)	[00:53,01:15]	00:57 (00:25)	[00:45,01:10]	*p* = 0.40*	0.13
	00:32–02:12		00:25–01:55			
Wake bouts	40.49 (14.46)	[34.65,46.33]	33.12 (9.36)	[28.47,37.78]	*p* = 0.05	0.61
	20–73		16–50			
Mobile minutes	44.81 (17.89)	[37.59,52.04]	34.75 (13.08)	[28.24,41.25]	*p* = 0.04	0.64
	20–85		15–67			
Fragmentation Index %	31.13 (10.16)	[27.03,35.23]	25.24 (8.19)	[21.17,29.32]	*p* = 0.04	0.64
	15–51		9–41			
Central Phase Measure	276.33 (84.87)	[242.05,310.61]	246.60 (82.43)	[205.60,287.59]	*p* = 0.25	0.60
	89.30–423.28		130.76–461.76			
**Sleep profile weekend**						
*N*	26		18			
TST (hh:mm)	06:39 (01:39)	[05:58,07:20]	06:30 (01:17)	[05:51,07:09]	*p* = 0.83*	0.03
	02:43–09:16		02:47–08:02			
Bed time (hh:mm)	01:37 (01:48)	[00:52,02:22]	01:40 (01:50)	[00:45,02:35]	*p* = 0.93	0.03
	20:51–04:15		22:45–06:40			
SOL (hh:mm)	00:37 (00:43)	[00:19,00:55]	00:30 (00:26)	[00:17,00:44]	*p* = 0.52	0.2
	00:01–03:34		00:01–01:39			
Waketime (hh:mm)	09:19 (01:44)	[08:36,10:02]	09:08 (00:59)	[08:38,09:38]	*p* = 0.67	0.13
	5:18–12:29		07:47–11:28			
Night wake (hh:mm)	01:07 (00:36)	[00:51,01:22]	00:56 (00:23)	[00:45,01:08]	*p* = 0.28	0.36
	00:14–02:35		00:27–01:39			
Wake bouts	41.96 (15.60)	[35.52,48.40]	35.46 (11.91)	[29.54,41.39]	*p* = 0.13	0.47
	16–79		19–56			
Mobile minutes	46.58 (23.08)	[37.05,56.11]	35.57 (14.81)	[28.21,42.94]	*p* = 0.08*	0.27
	12–113		19–65			
Fragmentation Index %	32.05 (10.58)	[27.68,36.42]	25.69 (7.79)	[21.81,29.56]	*p* = 0.03	0.68
	14–63		11–46			
Central Phase Measure	326.20 (89.60)	[289.21,363.18]	324.78 (78.23)	[285.87,363.68]	*p* = 0.42*	0.51
	87.50–473.25		201.40–499.80			
**Cognitive Function**						
*N*	27		18			
FSIQ	95 (11.80)	[90.33,99.67]	107.22 (9.55)	[102.48,111.97]	*p* < 0.001	1.14
	77–123		89–130			
VCI	101.22 (14.20)	[95.61,106.84]	107.67 (10.65)	[102.37,112.96]	*p* = 0.09	0.51
	74–138		89–130			
PRI	94.60 (10.05)	[90.62.98.57]	101.50 (9.96)	[96.55,106.45]	*p* = 0.03	0.69
	77–111		82–117			
WMI	97.07 (13.71)	[91.65,102.50]	108 (14.14)	[100.97,115.03]	*p* = 0.01	0.78
	74–136		89–136			
PSI	90.56 (13.3)	[85.29,95.82]	106.78 (10.38)	[101.62,111.94]	*p* < 0.001*	−0.59
	59–131		92–122			
**Cortisol**						
*N*	21		14			
Raw morning cortiosl	5.10 (4.10)	[3.23,6.96]	7.56 (5.03)	[4.65,10.46]	*p* = 0.16*	0.49
	0.89–13.48		1.38–16.39			
Diurnal cortisol	0.62 (0.61)	[0.34,0.9]	0.44 (0.43)	[0.2,0.69]	*p* = 0.21*	0.44
ratio	0.09–3		0.7–1.49			

*Note.* C= Controls, FSIQ= Full Scale IQ, PSI= Proxessing Speed Index, RRI = Perceptual Reasoning Index, SCD= Sickle cell disease, SCD + C= Comparison between SCD and controls, SES= Socioeconomic status, SOL= Sleep onset latency, TST = Total sleep time, VCI= Verbal Comprehension Index, WMI= Working Memory Index. Results are given in mean (±SD) and range.

*p value for Mann–Whitney *U* test.

### 3.2 Sleep profiles

Objective sleep profiles were measured using Actigraphy (Motionware 8). All forty-four individuals wore the Actiwatch between 5- 7 nights (*N*_SCD_ = 26, *N*_male_ = 10, Mean_age_ = 19.41 ± 5.24, range = 9–29 years; *N*_Control_ = 18, *N*_male_ = 5, Mean_age_ = 19.43 ± 3.99, range = 11–25 years). Only one participant did not wear the watch during the weekend. No significant differences between the groups were shown for TST, bedtime, Sleep Onset Latency (SOL), waketime and awakening (*p* > 0.05, Table 1). Significant differences between the groups were shown for wake bouts, mobile minutes, and fragmentation index (FI), with SCD participants experiencing lower sleep quality (i.e., more fragmented sleep) at night (Table 1).

After controlling for age, sex and SES, differences remained significant for mobile minutes (*F*(1,40) = 4.93, *p* = 0.032) and FI (*F*(1,40) = 4.69, *p* = 0.036), with a trend for a difference in wake bouts (*F*(1,40) = 3.55, *p* = 0.067). The majority of actigraphy measures also revealed no sex differences in SCD or control participants. Sex differences were only observed for mobile minutes (Mean♀ = 32.25 ± 8.27 vs. Mean ♂ = 42.90 ± 8.27, *p* = 0.049) in controls, with wake bouts (Mean♀ = 32.36 ± 8.45 vs. Mean ♂ = 38.1 ± 3.28, *p* = 0.055) in controls reaching near significance. At the weekend both groups slept more, had later bed- and wake times with slightly increased sleep disturbances compared to the week (Table 1). Interestingly, SOL was lower at the weekend for SCD participants compared to the week.

Greater nighttime activity (i.e., mean activity per epoch at night for 7 days), as measured from raw actigraphy data, was observed for SCD compared to controls (SCD: Mean = 6.42 ± 2.60, 95% CI [4.26, 6.81] range = 2.87–12.20 vs. controls: Mean = 5.54 ± 2.56, 95% CI [5.38, 7.47], range = 2.03–13, *p* > 0.05, *d* = 0.34) (Fig. 1). Mean day and night activity for each group is shown in Supplementary Fig. 2. There were no significant sex differences observed in either group (*p* > 0.05).

### 3.3 Cognitive profiles

There were significant differences between groups measures for FSIQ, WMI, PRI, and PSI (Table 1). The trend-level differences in VCI (Table 1) disappeared after controlling for age, sex and SES although significant differences remained for FSIQ (*F*(1,40) = 12.92, *p* = 0.001), PRI (*F*(1,40) = 4.84, *p* = 0.034), WMI (*F*(1,40) = 6.73, *p* = 0.013) and PSI (*F*(1,40) = 19.57, *p* < 0.001).

After controlling for PSI, all significant differences were abolished. There were sex differences in individuals with SCD for PSI only (Mean♀ = 95.50 ± 11.70 vs. Mean ♂ = 83.10 ± 13.25; *U* = 119.5, *z* = 2.12, *p* = 0.036). There were no significant differences in cognitive scores as a function of sex in controls.

### 3.4 Cortisol profiles

**Fig. 2:**
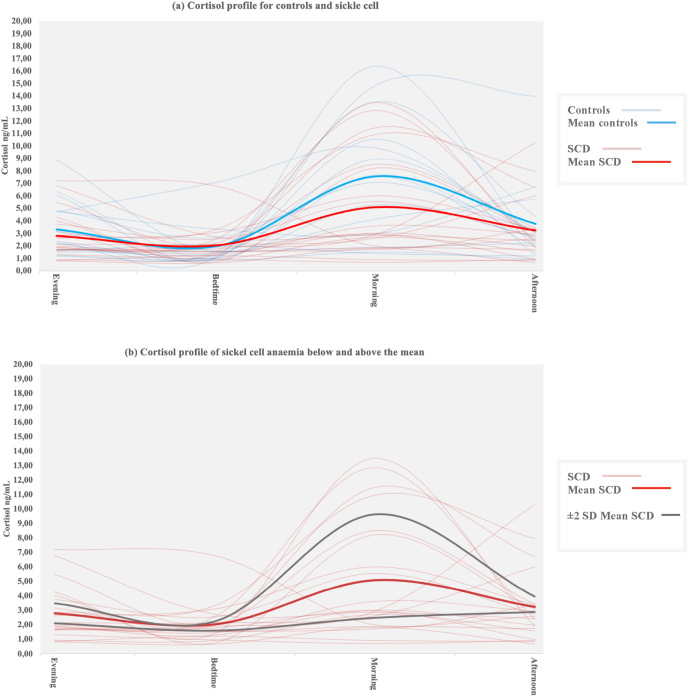
Cortisol profile in sickle cell disease and controls. Fig. 2(a): Cortisol profile for controls and sickle cell disease. Fig. 2(b): Cortisol profile for sickle cell disease. Note. FSIQ= Full Scale IQ, PRI= Perceptual Reasoning Index, PSI= Processing Speed Index, SCD= Sickle cell disease, VCI= Verbal Comprehension Index, WMI= Working Memory Index.

Thirty-six complete cortisol samples were analysed (*N*_SCD_ = 21, *N*_male_ = 7, Mean_age_ = 19.94 ± 4.80, range = 11.10–29.4 years; *N*_control_ = 15, *N*_male_ = 5, Mean_age_ = 19.02 ± 3.77, range = 11–24.8 years). There were no significant differences in cortisol measures as a function of sex in either group (*p* > 0.05). In terms of hypothesis (1) participants with SCD had lower raw morning cortisol and higher diurnal cortisol ratio (DCR) than controls (Table 1). Although the differences were not significant, the results are indicative of a group mean difference with medium effect size. Fig. 2(a) visually depicts the different cortisol profiles, showing normal cortisol profiles (NCP: N_SCD_ = 8, N_controls_ = 7) and flat cortisol profiles (FCP: N_SCD_ = 13, N_controls_ = 8) in SCD and controls. Morning cortisol was lower in SCD and controls with FCP compared to NCP (SCD: Mean_FCP_ = 2.31 ± 0.78 vs. Mean_NCP_ = 9.62 ± 3.01, *p* < 0.001 and Controls: Mean_FCP_ = 3.98 ± 2.49 vs. Mean_NCP_ = 11.67 ± 3.29, *p* < 0.001). DCR was higher in SCD and controls with FCP compared to NCP (SCD: Mean_FCP_ = 0.84 ± 0.69 vs. Mean_NCP_ = 0.26 ± 0.15, *U* = 7, *z* = −3.26, *p* < 0.001 and Controls: Mean_FCP_ = 0.61 ± 0.46 vs. Mean_NCP_ = 0.20 ± 0.23, *U* = 7, *z* = −2.43, *p* = 0.014). Fig. 2(b) shows differences in cortisol profile for participants with SCD only, based on their individual group mean.

#### 3.4.1 Cortisol and sleep

For hypothesis (2), sleep profiles of individuals with SCD and controls were compared with cortisol values. After controlling for age, sex, and SES, significant correlations for individuals with SCD were observed for morning cortisol and waketime (*r*(16) = −0.50, *p* = 0.033). Near significance was reached for total sleep time (*r*(16) = −0.45, *p* = 0.064) and central phase measure (*r*(16) = −0.45, *p* = 0.061) Near significance was reached for DCR and waketime (*r*(16) = 0.43, *p* = 0.072) and central phase measure (*r*(16) = 0.45, *p* = 0.058): After controlling for age, sex, and SES significant correlations for controls were observed for DCR and total sleep time (*r*(10) = 0.70, *p* = 0.013) only.

Individual group differences showed that those with a FCP, defined from the group mean, had longer total sleep time, later bed- and waketimes, experienced more night wakings, mobile minutes and a greater central phase measure compared to those with a NCP (Table 2) with moderate – large effect size (Fig. 2), in both groups. The results remained significant for waketime after controlling for age, sex and SES (Waketime: *F*(3,29) = 3.84, *p* = 0.02, ηp^2^ = 0.28) with a trend remaining for central phase measure: *F*(3,29) = 2.66, *p* = 0.07, ηp^2^ = 0.22).

**Table 2:** Comparisons between cortisol profiles for sleep and cognitive profiles in SCD and controls.

Variable	**SCD**				**Controls**				**Group difference**		
	Normal Cortisol Profile	Flat Cortisol Profile	*p*	*Effect size*	Normal Cortisol Profile	Flat Cortisol Profile	*p*	*Effect size*	*F*	*p*	*ηp2*
CI 95%	N = 8	N = 13			N = 7	N = 8					
**Sleep profile**											
TST (hh:mm)	06:11 (00:56)	06:42 (01:03)	*p* = 0.37*	0.52	05:55 (00:43)	06:23 (01:06)	*p* = 0.35	0.50	1.09	*p* = 0.37	0.09
	[05:24,06:58]	[06:04,07:21]			[05:15,06:35]	[05:27,07:19]					
Bed time (hh:mm)	00:47 (01:03)	01:35 (01:08)	*p* = 0.12	0.73	00:44 (00:57)	01:25 (01:28)	*p* = 0.30	0.55	1.27	*p* = 0.30	0.11
	[23:53,01:40]	[00:54,02:17]			[23:51,01:38]	[00:11,02:39]					
SOL (hh:mm)	01:07 (01:00)	00:36 (00:21)	*p* = 0.34*	0.69	00:46 (00:32)	00:26 (00:27)	*p* = 0.07*	0.64	1.86	*p* = 0.16	0.15
	[00:16,01:57]	[00:22,00:49]			[00:16,01:16]	[00:03,00:49]					
Waketime (hh:mm)	07:56 (01:25)	09:26 (01:10)	***p* = 0.03**	1.16	07:32 (00:42)	08:41 (01:26)	***p* = 0.07**	1.02	4.56	***p* = 0.01**	0.30
	[06:44,09:08]	[08:44,10:09]			[06:53,08:12]	[07:29,09:54]					
Night wake (hh:mm)	00:56 (00:20)	01:07 (00:31)	*p* = 0.55*	0.42	00:52 (00:07)	01:01 (00:29)	*p* = 0.44	0.43	0.57	*p* = 0.64	0.05
	[00:39,01:13]	[00:47,01:26]			[00:45,00:58]	[00:36,01:25]					
Wake bouts	37.19 (9.09)	42.55 (15.30)	*p* = 0.33	0.43	34.44 (6.12)	34.61 (8.84)	*p* = 0.97	0.02	1.17	*p* = 0.34	0.10
	[29.59,44.78]	[33.30,51.79]			[28.78,40.10]	[27.22,42,00]					
Mobile minutes	41.19 (16.60)	45.74 (16.74)	*p* = 0.55	0.27	33.84 (7.06)	36.84 (13.87)	*p* = 0.60	0.27	1.20	*p* = 0.33	0.10
	[27.32,55.06]	[35.63,55.85]			[27.31,40.37]	[25.23,48.44]					
Fragmentation Index %	31.03 (12.60)	30.86 (7.22)	*p* = 0.94	0.02	26.43 (6.36)	24.96 (8.88)	*p* = 0.72	0.19	1.06	*p* = 0.38	0.10
	[20.50,41.55]	[26.50,35.30]			[20.55,32.30]	[17.54,32.39]					
Central Phase Measure	262.40 (69.80)	331.81 (58.04)	***p* = 0.04**	1.08	249.33 (46.31)	308.69 (82.23)	*p* = 0.11	0.89	3.43	***p* = 0.03**	0.24
	[204.05,320.75]	[296.73,366.88]			[206.50,292.16]	[239.94,377.44]					
**Cognitive profile**											
FSIQ	93.75 (5.78)	93.38 (10.48)	*p* = 0.92	0.04	104.71 (12.57)	110.50 (6.82)	*p* = 0.31	0.59	7.21	***p* < 0.001**	0.40
	[88.92,98.58]	87.05,99.72]			[93.10,116.34]	[104.80,116.21]					
VCI	102.75 (8.66)	98.77 (10.60)	*p* = 0.21*	0.08	109.57 (12.89)	105.75 (10.60)	*p* = 0.34*	0.07	1.73	*p* = 0.18	0.14
	[95.51,109.99]	[92.36,105.17]			[97.65,121.50]	[96.90,114.61]					
PRI	97.13 (9.62)	91.69 (10.07)	*p* = 0.24	0.55	97.14 (11.64)	107.00 (6.05)	*p* = 0.08	1.1	4.23	***p* = 0.013**	0.28
	[89.06,105.19]	[85.60,97.78]			[86.38,107.91]	[101.94,112.06]					
WMI	96.00 (14.33)	95.00 (10.12)	*p* = 0.87	0.08	105.71 (15.50)	110.75 (15.72)	*p* = 0.54	0.32	2.89	***p* = 0.05**	0.213
	[84.02,107.98]	[88.89,101.11]			[91.38,120.05]	[97.61,123.89]					
PSI	91.25 (8.24)	88.46 (13.69)	*p* = 0.92*	0	102.29 (11.58)	112.25 (8.28)	*p* = 0.09	1	8.69	***p* < 0.001**	0.45
	[84.36,98.14]	[80.19,96.73]			[91.57,113.00]	[105.33,119.17]					

*Note.* C= Controls, FSIQ= Fulls Scale IQ, PRI= Perceptial Reasoning Index, PSI= Processing Speed Index, SCD= Sickle cell disease, SOL= Sleep onset latency, TST = Total sleep time, VCI= Verbal Reasoning Index, WMI= Working Memory Index. Results are given in mean (±SD). *p values for Mann–Whitney *U* test Effect size = Cohen's d.

#### 3.4.2 Cortisol and cognitive profiles

Participants with SCD who experienced a FCP scored non-significantly worse on all cognitive tests (Table 2). Interestingly controls who experienced a FCP did non-significantly better on tests measuring FSIQ, PRI, WMI and PSI, but not VCI (Table 2). Group differences as a function of cortisol profile were significant for FSIQ, PRI, WMI and PSI (Table 2). The largest group differences were observed for PSI and FSIQ.

After controlling for age, sex, and SES significant group differences were shown for FSIQ: *F*(3,29) = 5.56, *p* < 0.001, ηp^2^ = 0.53; VCI: *F*(3,29) = 3.37, *p* 0 0.012, ηp^2^ = 0.41, PRI: *F*(3,29) = 3.55, *p* = 0.009, ηp^2^ = 0.423 and PSI: (*F*(3,29) = 4.617, *p* = 0.002, ηp^2^ = 0.489, with a near-significance for WMI: *F*(3,29) = 2.18, *p* = 0.74, ηp^2^ = 0.31.

#### 3.4.3 Relationship between sleep, cognition, and cortisol

Group comparison showed significant differences and medium effect sizes for cortisol, cognition, and Fragmentation Index (FI, an indication for sleep quality). Therefore, we investigated a three-way moderation to understand the effect of raw morning cortisol (X) on cognition (Y) by group health (W), which is moderated by sleep quality (measured with FI) (Z), while controlling for age, sex, and SES. The overall model was significant for FSIQ: *R*^2^ = 0.57, *F*(10, 25) = 3.3, *p* = 0.01; VCI: *R*^2^ = 0.51, *F*(10, 25) = 2.63, *p* = 0.02, *p* = 0.02; PSI: *R*^2^ = 0.53, *F*(10, 25) = 2.84, *p* = 0.02, *p* = 0.02, and PRI*: R*^2^ = 0.59, *F*(10, 25) = 3.57, *p* = 0.005 but not for WMI: *R*^2^ = 0.37, *F*(10, 25) = 1.50, *p* = 0.20.

PRI was the only variable showing evidence for a three-way interaction (XWZ) between morning cortisol, group health and FI (*b*_*7*_ = 0.19, *t*(25) = 2.20, *p* = 0.038). The magnitude of the moderation by group health of the effect of morning cortisol on PRI depends on sleep quality, however, this “moderation of moderation” describes only 8% of the variance in PRI.

**Fig. 3:**
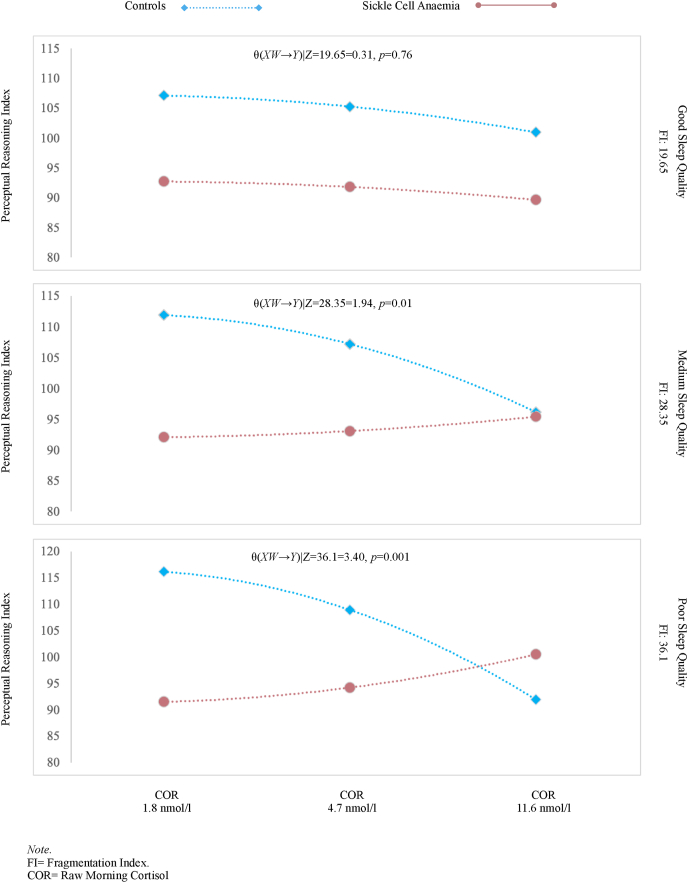
The effect of cortisol on perceptual reasoning index as a function of sleep quality in individuals with sickle cell disease and controls.

Among individuals with good sleep quality (i.e., low sleep fragmentation) (Z_FI_ = 19.65), the effect of morning cortisol on PRI was not significantly moderated by group health (SCD vs. controls) *θ*_*XW→Y*_ |(*Z*_*FI*_ = 19.65) = 0.31, *F*(1,25) = 0.09, *p* = 0.76. But among individuals with medium sleep quality (i.e., medium sleep fragmentation) *θ*_*XW→Y*_ |(*Z*_*FI*_ = 28.35) = 1.94, *F*(1,25) = 8.32, *p* = 0.01 and poor sleep quality (i.e., high sleep fragmentation) *θ*_*XW→Y*_ |(*Z*_*FI*_ = 36.10) = 3.40, *F*(1,25) = 13.47, *p* = 0.001, group health significantly moderated the effect of cortisol on PRI. The significant interaction between group health and morning cortisol is reached for those with a FI > 28.84. Below this value group health does not moderate the effect of morning cortisol on PRI. Visually depicted, individuals with SCD, who experienced low morning cortisol and all ranges of sleep quality (i.e., low – poor) scored worse on PRI compared to controls. Interestingly, SCD with high morning cortisol and poor sleep quality performed better on PRI compared to controls, who performed worse (Fig. 3).

Using DCR as an indication of the change in cortisol from morning to evening, the model was also significant for an effect on PRI *R*^2^ = 0.52, *F*(10, 25) = 2.68, *p* = 0.022, with a significant three-way interaction (XWZ) for PRI with DCR, group health and FI (*b*_*7*_ = −2.94, *t*(25) = -2.63, *p* = 0.01), but not for the other cognitive variables. The magnitude of the moderation by group health of the effect of DCR on PRI depends on sleep quality (FI), however, this “moderation of moderation” describes only 13% of the variance in support of PRI.

Among individuals with good sleep quality (i.e., low sleep fragmentation) (Z_FI_ = 19.65), the effect of DCR on PRI is not significantly moderated by group health (SCD vs. controls) *θ*_*XW→Y*_ |(*Z*_*FI*_ = 19.65) = 1.49, *F*(1,25) = 0.03, *p* = 0.88. But among individuals with medium sleep quality (i.e., medium sleep fragmentation) *θ*_*XW→Y*_ |(*Z*_*FI*_ = 28.35) = −24.1, *F*(1,25) = 8.58, *p* = 0.01 and poor sleep quality (i.e., high sleep fragmentation) *θ*_*XW→Y*_ |(*Z*_*FI*_ = 36.10) = −46.83, *F*(1,25) = 10.51, *p* = 0.003, group health significantly moderates the effect of DCR on PRI. The significant interaction between group health and DCR is reached for those with a FI > 25.27. Below this value group health does not moderate the effect of morning cortisol on PRI. Individuals with SCD, who experienced a high DCR/flat cortisol profile and high sleep fragmentation at night scored worse on PRI compared to controls. Interestingly, individuals with SCD with a normal cortisol profile performed slightly better on PRI despite high movement at night compared to controls, who performed worse. Interestingly, SCD with high morning cortisol and high FI performed better on PRI compared to controls, who performed worse.

## 4 Discussion

### 4.1 Sleep profiles

This is the first study to investigate the relationship between sleep, cortisol, and cognition in SCD. Our findings support the current literature in the understanding that individuals with SCD experience sleep disturbances (e.g., greater sleep fragmentation, mobile minutes and wake bouts at night as measured with actigraphy in our study). The current findings show that young people with SCD have shorter sleep duration than the recommendations given by the National Sleep Foundation [51], which was also reported by Ref. [9] who identified that 70% took more than 20 min to fall asleep and that 63% of preschool children, 40% of teens and 15% of young adults slept less than recommended at night. They also have longer sleep latency, experienced greater wake bouts, mobile minutes and fragmented sleep at night compared to controls.

The objective use of actigraphy confirmed previous findings showing that individuals with SCD experience greater sleep fragmentation and mobile minutes at night. It is possible that greater sleep fragmentation might not only be due to sleep apnoea and frequent awakenings at night, but also related to PLMS, shown to be high in this population [6]. Research in SCD identified 23.4% of children (mean age = 8.5 years) [52] and 70% of adults (23–41 years) to have a PLMS >5/h, with 36% of adults even showing a PLMS of >15/h [6]. Cabañas-Pedro and colleagues (2017) reported that severity of SCD was associated the occurrence of PLMS at night. Additionally, painful vaso-occlusive crises could further explain lower sleep quality in SCD, which was shown to reduce total sleep time in 12–64-year olds with SCD [53].

The link between sleep disorders and sleep disruptions are already shown in the general population [54]. It seems that sleep related problems are already present at early developmental stages in SCD [9]; however, unlike in typical development where sleep problems are transient, these appear to persist through the lifespan and might contribute to long-term health consequences. Future research needs to disentangle this relationship in SCD, using a longitudinal approach, with comparison to other clinical and healthy populations using mixed method approach to assess sleep patterns.

### 4.2 Cognitive profiles

It is well known that sleep is important for brain plasticity [55], especially to facilitate learning [56] at night. Daytime sleepiness, either because of disease related anaemic events and/or sleep loss, could exacerbate difficulties in cognitive functioning due to poorer attentional processing and memory consolidation of new information. Individuals with SCD often experience processing speed difficulties, which is needed to support other functions such as working memory. Our findings are in line with the previously reported cognitive difficulties observed between SCD and healthy controls.

However, we found a larger cognitive difference between SCD and controls for FSIQ (12 points) and PSI (16 points) than previously reported for FSIQ (7 points) [18] and PSI (9 points) [40] on the Wechsler Scales. The difference could be explained by the high prevalence of sleep disturbances (i.e., FI SCD 31% and Controls 25%) in our population, which is similar to other findings in children with SCD (mean age 12 years, FI 33%) experiencing problems in executive functioning [57]. People with SCD experience more sleep fragmentation compared to controls, which could be an indication of SDB albeit it was not examined here. Frequent arousals at night and exposure to hypoxia, with nocturnal oxygen desaturation, could be contributing factors to the observed cognitive difficulties in people with SCD. There is evidence that children with SCD (8-16-year-olds) and OSA may have lower executive functioning [17], with evidence for an effect of severe OSA on processing speed [16].

Interestingly, VCI showed the smallest differences between the groups. Although lower scores were observed for VCI in SCD, the majority scored in the normal range. Verbal comprehension develops considerably during childhood and adolescence and is also known as a “crystallised” ability. It tends to remain stable later in life but depends on acquired knowledge. It is now well established in developmental psychology that there is a critical period, during which environmental stimuli can significantly impact on a child's development. Could we not see a difference in VCI, because it might become apparent later in life? Perhaps crystallised abilities in SCD are more “protected” because the impact of sleep and health difficulties become apparent later in development. Therefore, we propose that the onset, duration, and severity of sleep disorders could play a significant role in the development of cognitive difficulties. However, this needs further exploration.

It is also important to take under consideration the large difference in processing speed between SCD and controls. Similar to our research a 10-point difference in processing speed was found [58]. The authors further suggest that individuals with SCD might perform equally well or similar on cognitive tasks compared to controls if their processing speed was considered [40].

Crucial for the development of new interventions such as individualising existing strategies and programmes. It is recommended here that additional time for homework, exams and breaks to rest as well as napping facilities ought to be considered to enhance learning and daytime functioning of young people from the early years.

### 4.3 Cortisol profiles

Cortisol's biological effects are generically metabolic, in that it increases fat catabolism, glycogen-glucose levels and energy production, alongside the specific brain and immune physiological responses. During the day cortisol levels fluctuate widely due to the numerous neuro-physiological and metabolic demands and stressors experienced. However, the ratio of an individual's waking and bedtime (i.e., diurnal cortisol ratio) should be an extreme and is the expected norm, which is a quantification marker and measure of neurophysiological stressors disrupting the diurnal metabolic norm of the sleeping and waking circadian rhythm. Thus, similar to studies examining cortisol profile in developmental disorder such as Williams Syndrome [45], we found that individuals with SCD experience a flattened cortisol profile/diurnal ratio compared to controls [26,27,30]. Later waketimes in the morning, and bedtimes after midnight were associated with lower diurnal variation in both groups. One explanation could be that lower sleep quality, either experienced through sleep fragmentation or shorter sleep duration, might dysregulate the HPA-axis, and thus cortisol secretion, as was recently shown in healthy adults by Vargas and colleagues (2020) [25]. However, the most direct explanation for the observed differences in cortisol profile is that individuals with SCD experience more emotional and physical stress related to their disease severity (i.e., frequent hospital visits, pain, and mental health problems). Thus, being confronted with stressful situations and not being able to manage these well could further dysregulate cortisol diurnal circadian rhythm, feeding back on the HPA-axis and therefore impairing child development at an early age with delayed growth, metabolic and altered immune response [59].

The current study has shown that sleep in SCD might be more fragmented because of increased movement at night. Therefore, cortisol diurnal circadian- HPA dysregulation might promote feelings of tiredness and poor concentration during the day, reducing learning capacities. Individuals with SCD who lacked diurnal variation performed worse on tests measuring cognitive functioning compared to SCD with normal cortisol profile, while it was the other way around for controls. Only VCI was 4 points lower in controls with flat a cortisol profile, performing still in the average range. Processing speed and perceptual reasoning skills showed the lowest scores in individuals with SCD and low diurnal variation. Neuronal activation in frontal and parietal lobes is important for attentional and complex thinking; however, these are disrupted with sleep deprivation, thus decreasing cognitive performance [11]. For example perceptual reasoning, important for complex abstract and novel thinking skills were shown to be lower in healthy children (mean age 9 years) with late and irregular bedtimes [60].

Our moderator analysis provides evidence that perceptual reasoning is worse in individuals with SCD who experience lower morning cortisol and poor sleep quality (i.e., Fragmentation Index, measured as mobile minutes by actigraphy). The effect of morning cortisol between SCD and controls on perceptual reasoning index was significant when there were a FI of >28.8% at night. Interestingly, our results revealed better perceptual reasoning performance in SCD who experienced higher morning cortisol despite low sleep quality, while our controls performed worse in the same category. This outcome was partially confirmed by Ref. [38]; who reported that higher cortisol during testing was associated with better perceptual reasoning scores in undergraduate students. One explanation could be that higher morning cortisol might be beneficial for SCD, who generally experience lower cortisol secretion. The authors also report that test anxiety had a negative impact on perceptual reasoning, which might explain the lower scores in controls. However, other factors could drive the relationship between sleep, cortisol, and cognition in SCD, such as prescribed medication, adaptation to stress and individual differences (i.e., compensatory mechanism allowing good performance on cognitive test despite sleep deprivation). Future studies need to investigate potential factors contributing to the altered cortisol response in SCD to formulate early interventions.

### 4.4 Limitations

There were some limitations to our study, which should be considered for future research. In this study we did not include polysomnography, the gold standard to measure sleep stages and sleep disorders, which can also accurately diagnose PLMS and OSA. Using Actigraphy on the ankle, with a trial-axial sensor could further help to detect PLMS more accurately. The collection of salivary cortisol, although clearly stated with instructions and pictures, was depending on the participant's reliability and accuracy. Although salivary cortisol is a more cost effective, acceptable, and simple measure to take, future studies could also examine cortisol levels in the blood night to understand its profile in relationship to sleep disturbances in SCD. Due to lack of access to medical records we were not able to assess potential relationships between cortisol and daily prescribed medication in SCD. For example, opioids can suppress periodic limb movement symptoms [61] and hydroxyurea alleviate symptoms of OSA in children with SCD [62]. All efforts in recruitment were made to invite individuals with SCD and to find age and ethnicity-matched healthy controls. However, this is a vulnerable group and we hope to invite more participants to our future research to look at developmental differences across a larger and wider age range. Although the WISC-IV and WAIS-IV are widely used, a recent commentary has noted that these tests underestimate the influence of general and fluid intelligence on the different cognitive domains [63] Therefore, future research should replicate this finding with the current version of the WISC-V and WAIS-V, as well as using other cognitive assessments that measure the same cognitive domains in SCD.

### 4.5 Conclusion

Individuals with SCD experience sleep disturbances, in association with altered cortisol profiles and cognitive difficulties, all of which may affect later social and economic mobility. These disturbances may also have negative impact on optimal learning and daytime functioning for children and become challenging in employment settings. Interventions need to be targeted in the early years of the developmental stages before health-related problems could manifest itself and cause long-term difficulties. More research is needed to explore the role that sleep plays in SCD, but our data suggest that it is an important one, understanding of which could also improve health and well-being in this population.

## Disclosure statement

FJK has received fees for work outside this study on advisory boards for Johnson and Johnson, Shire, Novartis, Bluebird Bio, Global Blood Therapeutics and BIAL/Eisai. The remaining authors have no conflicts of interest to disclose.

## Funding

MK and HS were funded by 10.13039/501100000317Action Medical Research (GN2509). This research was supported by the 10.13039/501100000272NIHR Great Ormond Street Hospital 10.13039/100014461Biomedical Research Centre (GOSH BRC: IS-BRC-1215-20012 Training Theme). The BRC had no role in the design and conduct of the study. The views expressed are those of the author(s) and not necessarily those of the NHS, the NIHR or the Department of Health. DD was funded by John and Lorna Wing Foundation.

## Author contributions

Data curation, M.K., H.S., C.B., S.S.; formal analysis, M.K.; funding acquisition, F.J.K, DD.; methodology, M.K., F.J.K., DD; supervision, F.J.K. and D.D.; visualisation, M.K.; writing—original draft, M.K. writing—review and editing, M.K., F.J.K., D.D, R.I., H.S., E.H. All authors have read and agreed to the published version of the manuscript.
