# Supplementary material for: Exploring the relationship of sleep, cognition, and cortisol in sickle cell disease
Source: Compr Psychoneuroendocrinol. 2022 Mar 4;10:100128. doi: 10.1016/j.cpnec.2022.100128 (PMC9216257; doi:10.1016/j.cpnec.2022.100128)
Supplement: Multimedia component 1 [file mmc1.pdf]

Supplementary information

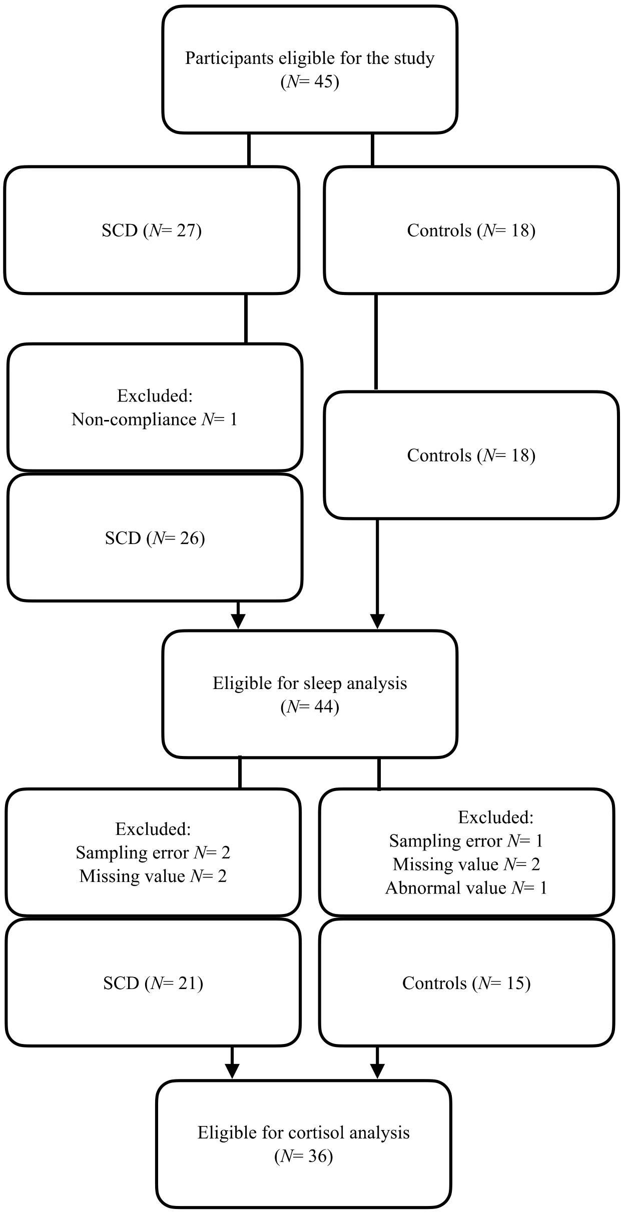

Supplementary Figure 1.
Flowchart.

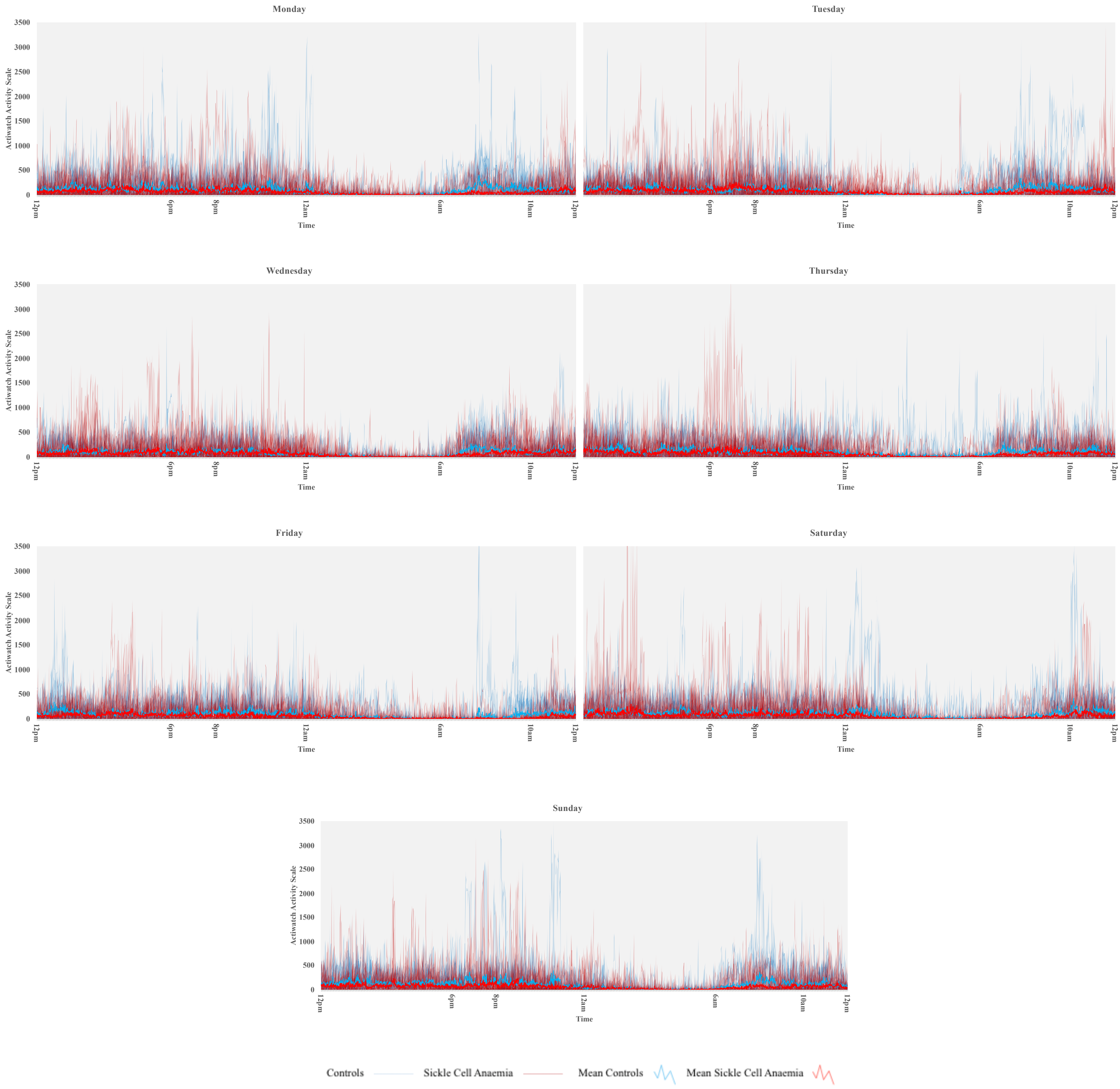

Supplementary Figure 2.

Actiwatch activity for each day of the week for sickle cell disease and controls.
